# Electrochemical and Kinetic Performance of Low-Cobalt and Cobalt-Free Rare-Earth AB_5_-Type Hydrogen Storage Alloys

**DOI:** 10.3390/ma18143317

**Published:** 2025-07-14

**Authors:** Yingying Shen, Fengji Zhang, Hengyu Ma, Yun Zhao, Yong Wang, Xinfeng Wang, Xiuyan Li, Youcheng Luo, Bingang Lu

**Affiliations:** 1 School of Materials Science and Engineering, Lanzhou University of Technology, Lanzhou 730050, China; shenyy@lut.edu.cn (Y.S.); 15719132908@163.com (F.Z.); 13294580346@163.com (H.M.); 13165790807@163.com (Y.Z.); wangyong5202025@163.com (Y.W.); 2 Gansu Rare Earth New Material Limited-Liability Company, Baiyin 730900, China; lzwxf5201314@163.com (X.W.); 15193097263@163.com (X.L.); luoyc1217@163.com (Y.L.); 3 State Key Laboratory of Ni &Co Associated Minerals Resources Development and Comprehensive Utilization, Jin Chuan Group Co., Ltd., Jinchang 737104, China

**Keywords:** low cobalt, cobalt free, hydrogen storage alloys, electrochemical performance

## Abstract

To address the high cost of cobalt in rare-earth hydrogen storage alloys, this study developed cost-effective low-cobalt and cobalt-free AB_5_-type alloys. The results demonstrate that all synthesized alloys displayed a single-phase LaNi_5_ structure possessing a homogeneous elemental distribution. Low-cobalt (La, Ce) (Ni, Co, Mn, Al)_5_ alloy 4SC and cobalt-free (La, Ce) (Ni, Mn, Al)_5_ alloy 7D exhibited similarly excellent electrochemical performance, including high discharge capacity, long cycle life, and superior high-rate discharge (*HRD*) capability. In addition, the kinetic test results show that the exchange current densities of these two alloys were quite similar, measuring 302.97 mA g^−1^ and 317.70 mA g^−1^, respectively. However, the hydrogen diffusion coefficient of 7D was significantly higher than that of 4SC, reaching 9.45 × 10^−10^ cm^2^ s^−1^, while that of 4SC was only 5.88 × 10^−10^ cm^2^/s. This work establishes a theoretical foundation for industrial-scale and cost-effective AB_5_-type hydrogen storage alloys, offering significant commercial potential.

## 1. Introduction

To strengthen the market competitiveness of Ni/MH batteries, lower costs and optimize efficiency are essential to the development of low-cobalt and cobalt-free rare-earth AB_5_-type hydrogen storage alloys [1,2,3,4,5,6]. The electrochemical capacity, long cycle life, good overcharge–overdischarge characteristics, and excellent environmental compatibility of rare-earth AB_5_-type hydrogen storage alloys make them widely applicable as anode materials in nickel/metal hydride (Ni/MH) batteries [7,8]. The A-side elements are composed of rare-earth elements featuring large atomic radius and heat-producing properties, capable of absorbing substantial amounts of hydrogen to form stable hydrides; they include La, Ce, Pr, Nd, and others. The B-side elements are metallic elements with smaller atomic radius and heat-absorbing properties that have low hydrogen affinity (so hydrogen can easily move); they include Mn, Fe, Co, Ni, Cu, Al, and others [9]. Cobalt (Co) strengthens the alloy’s cycle stability [10], boosts micro-toughness, decreases volume expansion following hydrogen desorption, curbs alloy pulverization, and restrains the dissolution of elements such as Mn and Al, thus decelerating the alloy’s corrosion rate during charge–discharge cycles and extending its cycle life. However, the strong interaction between Co and hydrogen is unfavorable for the high-rate discharge of the alloy [11].

Being a strategically scarce resource, the price of Co has risen sharply over the past few years, increasing the cost of rare-earth hydrogen storage alloys and making Ni/MH batteries expensive. Based on the composition of typical AB_5_ hydrogen storage alloys, it is calculated that Co accounts for 40–50% of the total material cost. Compared with Co and Ni, the low-cost Fe represents another 3d transition metal, which demonstrates comparable cycle stabilization within AB_5_-type hydrogen storage alloys [12,13,14,15,16]. Since Cu, Fe, and Ni are adjacent elements in the same period of the periodic table, their chemical properties are similar, and the addition of Cu particularly enhances the resistance to pulverization of the alloy. Tliha, M. [17] systematically studied the effect of the Fe element on the electrochemical properties of the alloy LaNi_3.55_Mn_0.4_Al_0.3_Co_0.6_Fe_0.15_ under low-Co conditions. It was found that with the increase in the number of charge–discharge cycles, the corrosion resistance of the alloy increased, and only microcracks occurred in the metal hydride alloy powder, improving its cycle stability. Due to the particles formed by microcracks, the actual surface area of the alloy increased, thereby affecting its kinetic properties. A study on the MlNi_3.5_Co_0.7−7x_Cu_8x_Al_0.8−x_ alloy [18] found that an appropriate amount of Cu can reduce the expansion ratio of the alloy after hydrogen absorption, lowers its microhardness, and is beneficial to improving the alloy’s resistance to powdering. Mn is essential to reducing the hydrogen absorption/desorption plateau pressure. Al is present in almost all commercial AB_5_-type electrodes, and even small amounts can significantly reduce lattice expansion and corrosion [19].

In this study, relatively inexpensive elements such as Cu, Fe, and Mn are used to replace Co on the B-side of AB_5_-type hydrogen storage alloys. Five low-cobalt and cobalt-free hydrogen storage alloys were prepared via the vacuum strip casting and heat treatment processes by a rare earth company. The crystal structure and electrochemical properties of the alloys were investigated for exploring their industrial applications. The results indicate that the alloys under investigation exhibit commercial value and meet various industrial requirements.

## 2. Experimental Methods

### 2.1. Preparation of Alloys

To systematically explore low-cobalt and cobalt-free rare-earth hydrogen storage alloys, the electrochemical and kinetic characteristics of these five alloys were examined with different doping elements and contents under rapid-solidification conditions. The compositions are (La, Ce) (Ni, Co, Mn, Al)_5_, (La, Ce) (Ni, Mn, Al)_5_, and three variants of (La, Ce) (Ni, Mn, Al, Cu, Fe)_5_, which are designated as 4SC, 7D, 7L, 8A, and 8B.

The alloys were prepared using a strip casting furnace (VGI-50SC, Shenyang Guangtai vacuum equipment Co., Ltd, Shenyang, China). Prior to the melting process, the furnace underwent initial evacuation to 3 Pa, followed by argon introduction. The arc heating method was employed to melt the metals with purity greater than 99 wt%. Then, the alloy melt was subsequently poured onto a high-speed-rotating water-cooled copper roller for rapid solidification, forming thin sheet samples with an average thickness of about 0.2 mm. Next, 3 wt% La, Ce, and Al and 5 wt% Mn were added to the starting material to take into account the material loss generated during arc melting.

To achieve uniform alloy composition, refined grains, and the elimination of casting defects, the prepared sheet samples were heat-treated at 1213 K for 6 h. Finally, the samples were mechanically crushed and sieved through a 200-mesh sieve, with an appropriate amount of powder taken for testing.

### 2.2. Structural Characterization

X-ray diffraction (XRD; PANalytical Empyrean, Malvern Panalytical, Malvern, UK; Cu Kα radiation) was utilized to characterize the alloy microstructures. The wavelength was 0.15406 nm, the scanning range was 10° to 90°, and the scanning rate was 2° per minute. The collected data were analyzed using Jade 6.5 software equipped with the PDF2004 crystal database for phase identification. Rietveld full-pattern fitting was performed to determine the phase composition and unit cell parameters. A weighted full-pattern factor *R*_wp_ ˂ 10 indicates a reliable result.

The alloy block was embedded with polymethyl methacrylate (PMMA), then ground sequentially with sandpaper of different grit sizes (from low to high magnification), and polished using polishing paste with varying particle sizes, yielding qualified test samples with a flat, bright, and scratch-free surface ready for testing. The microstructural morphology of the samples was obtained using the Quanta FEG450 field-emission scanning electron microscope (FE-SEM) (purchased from FEI Company, Shanghai, China) via backscattered electron (BSE) imaging. By analyzing brightness contrast and combining it with EDS (Energy-Dispersive X-ray Spectroscopy) analysis, the phase composition of the alloys was determined. With a magnification of 800× and an accelerated voltage of 20 kV, the morphology and compositional information of the alloy were obtained.

### 2.3. Pressure–Composition–Temperature (PCT) Tests

The Pressure–Composition–Temperature (PCT) isotherms were determined via a Suzuki Sieverts-type apparatus (Suzuki, Japan) at 318 K. The alloy powders (below 200 mesh) were activated at 523 K to ensure complete activation within a short period.

The inclination of the plateau is primarily attributed to lattice defects and related factors. The inclination of the plateau can be quantified using the slope value, and the plateau slope (*F*) is determined by the following equation [20]:

(1) $$F=\frac{ln\frac{p_{75\%}}{p_{25\%}}}{75\%C_{max}-25\%C_{max}}$$

In the equation, *P*_75%_ and *P*_25%_ represent the hydrogen pressure values corresponding to the hydrogen contents at 75% *C*_max_ and 25% *C*_max_ on the desorption curve, respectively.

The discrepancy between hydrogen absorption and desorption plateau pressures indicates the degree of micro-stress within the alloy lattice. The discrepancy of the absorption and desorption plateau pressures can be measured via hysteresis, and the hysteresis coefficient (*H*_f_) is calculated using the following formula [21]:

(2) $$H_{f}=ln(\frac{p_{abs}}{p_{des}})$$

In the equation, *P*_abs_ and *P*_des_ represent the pressure values corresponding to the mid-values at the hydrogen absorption and desorption plateaus, respectively.

### 2.4. Electrochemical Measurements

The electrochemical performance was assessed via a LANHE battery test system (CT3002A, purchased from Wuhan Landun Electronic Co., Ltd., Wuhan, China) employing a three-electrode setup comprising a metal hydride working electrode, a Hg/HgO reference electrode, and a sintered Ni(OH)_2_/NiOOH counter electrode within a 6 mol·L^−1^ KOH aqueous solution. The hydrogen storage alloy and carbonyl nickel powder were homogeneously blended in a 1:4 mass ratio and then compacted into pellets 10 mm in diameter and 1 mm in thickness under 10 MPa pressure with a hydraulic pressing machine (HY-24, Shanghai Keheng Industrial Development Co., Ltd., Shanghai, China). Electrochemical measurements were performed at ambient temperature (298 K).

The electrode was subjected to charging over a 6 h duration at a current density of 0.2 C (60 mAh g^−1^) and subsequently discharged at a 0.2 C rate with a termination voltage of 0.65 V (vs. Hg/HgO reference electrode) to facilitate activation. The electrochemical capacity of the electrode was determined using the active material mass per gram. The standard capacity test is conducted by defining the total discharge capacity at the 8th cycle as the test result. For cycles 1–2, the alloy was charged at a 0.2 C-rate for 6 h; for cycles 3–8, charging was performed at 1 C (300 mAh g^−1^) for 72 min. Discharge was carried out stepwise at a 2 C rate, a 1 C rate, and a 0.4 C rate, with a cutoff voltage of 0.65 V. Rapid activation is applied to evaluate the alloy’s conventional capacity, represented by the average capacity value. The electrode was charged and discharged at a current density of 1 C (300 mAh g^−1^) to evaluate the cycling stability. High-rate discharge tests were also conducted by charging the electrode at 1 C, followed by discharging at 1 C, 3 C, 5 C, and 10 C, under a termination voltage of 0.65 V.

High-rate discharge (*HRD*) is determined via the following equation:

(3) $$HRD=\frac{C_{i}}{C_{i}+C_{60}}$$

In the equation, *C*_i_ represents the discharge capacity under a current density of *i* (*i* = 1 C, 3 C, 5 C, and 10 C), and *C*_60_ denotes the remaining discharge capacity under a current density of 0.2 C.

### 2.5. Electrochemical Kinetic Measurements

Electrochemical kinetic tests were conducted with an electrochemical workstation (CHI660E, Shanghai CH Instruments Co., Ltd., Shanghai, China). The exchange current density (*I*_0_) was derived using the linear polarization curve through potential scanning in the range of equilibrium potential *E*_eq_ ± 6 mV and a sweep rate of 1 mV s^−1^. The value of *I*_0_ was determined using the slope of the curve [22]:

(4) $$I_{0}=\frac{RT}{nF}\frac{I(t)}{\eta (t)}$$

In the equation, *T* denotes the absolute temperature, *R* stands for the molar gas constant, *F* symbolizes Faraday’s constant, *η*(t) represents the overpotential, and *I*(t) designates the polarization current. The hydrogen diffusion coefficient (*D*) is derived using the chronopotentiometric curve, via a potential step applied at *E*_eq_ + 0.6 V and a duration of 3000 s. It can be calculated using the following formula:

(5) $$logi=\log\left(\frac{6FD\left(C_{0}-C_{s}\right)}{{da}^{2}}\right)-(\frac{\pi^{2}}{2.303})(\frac{D}{a^{2}})t$$

In the equation, *D* denotes the hydrogen diffusion coefficient (in units of cm^2^ s^−1^), *a* symbolizes the radius of the alloy particles (in cm), *i* designates the diffusion current density (A g^−1^), *C*_0_ represents the initial hydrogen concentration in the alloy (mol cm^−3^), *C*_s_ denotes the hydrogen concentration on the surface of the alloy particles (mol cm^−3^), *d* stands for the density of the alloy (g cm^−3^), and *t* corresponds to the discharge time (s). The corrosion current density (*i*_corr_) was derived via the extrapolation of the Tafel curve. The scanning potential was adjusted to the equilibrium potential *E*_eq_ ± 0.25 V at a scan rate of 5 mV s^−1^. The corrosion rate of the metal can be determined via the following equation [23]:

(6) $$\eta_{c}=-\beta_{c}log(i_{c}/i_{corr})$$

In the equation, *i*_corr_ represents the corrosion current density (μA cm^−2^), *η*_c_ denotes the cathodic overpotential (mV), *i* is the measured current density (μA cm^−2^), and *β*_c_ is the cathodic Tafel slope (mV/decade).

Electrochemical impedance spectroscopy (EIS) measurements were performed on the alloy in the frequency range spanning 10^−4^ Hz to 10^4^ Hz, using a potential perturbation magnitude of 5 mV. The EIS equivalent circuit comprises bulk impedance (*R*_b_), contact capacitance (*Q*_ol_), resistance (*R*_ol_), double-layer capacitance (*Q*_dl_), and charge transfer resistance (*R*_ct_) [24,25]. Before the kinetic tests, the exchange current density, Tafel curve, and electrochemical impedance spectra were measured, and the current was discharged to 50% of the electrode capacity (50% discharge depth, DOD). Before the chronopotentiometric tests, the electrode was fully charged, and all tests were performed after activation. Prior to electrochemical and kinetic performance testing, all alloy electrodes were subjected to six charge–discharge cycles under the same charge–discharge protocols as activation to ensure consistency.

## 3. Results and Discussion

### 3.1. Crystal Structures

**Figure 1:**
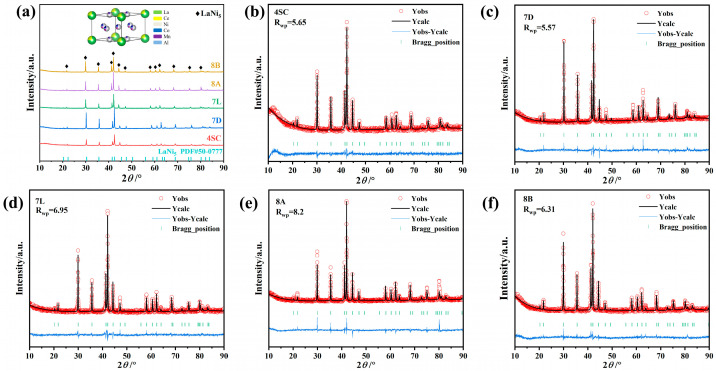
XRD spectra and Rietveld refinement plots of the AB_5_-type hydrogen storage alloys: (**a**) XRD spectra of the alloys and (**b**–**f**) Rietveld full-profile fitting of XRD patterns for hydrogen storage alloys.

The XRD spectra and Rietveld refinement plots of the hydrogen storage alloys are presented in Figure 1a–f.

Figure 1a displays the XRD diffraction patterns of the five hydrogen storage alloys, with the inset illustrating the crystal structure of alloy 4SC. It is evident that all alloys exhibit a single-phase CaCu_5_-type of LaNi_5_ structure (space group: P6/mmm), indicating that the doping of different metal atoms has not altered the alloys’ crystal structure. Figure 1b–f show the Rietveld refinement plots of the XRD patterns for the alloys. From the fitting results, it is evident that the refined curves closely match the experimental measurements, demonstrating the validity of the refinement outcomes. The crystallographic parameters obtained from Maud fitting are summarized in Table 1. As shown in Table 1, among the three alloys with identical compositions (7L, 8A, and 8B), alloy 8A exhibits the maximum unit cell volume. Between alloys 4SC and 7D, alloy 7D has the minimum unit cell volume. Furthermore, comparing the unit cell volumes across all alloys, the volume of alloy 8A is the largest, while that of alloy 7D is the smallest. According to the atomic radii of the elements, where r_La_ (0.187 nm) > r_Ce_ (0.183 nm) > r_Al_ (0.143 nm) > r_Fe_ (0.140 nm) > r_Mn_ (0.136 nm) > r_Cu_ (0.128 nm) > r_Co_ (0.125 nm) > r_Ni_ (0.124 nm), it can be concluded that elements with larger atomic radii increase the unit cell size during substitution. Alloy 8A, with higher contents of La and Al, exhibits a larger unit cell volume compared with alloys 7L and 8B. In contrast, alloys 4SC and 7D show a smaller unit cell volume, with alloy 7D being the smallest due to the doping of Co. Furthermore, as the anisotropic c/a ratio increases, hydrogen atoms tend to occupy the interstitial sites within the CaCu_5_ structure, leading to the formation of metal hydrides, thereby significantly reducing stress concentration and pulverization in the alloys [16,26,27,28].

**Figure 2:**
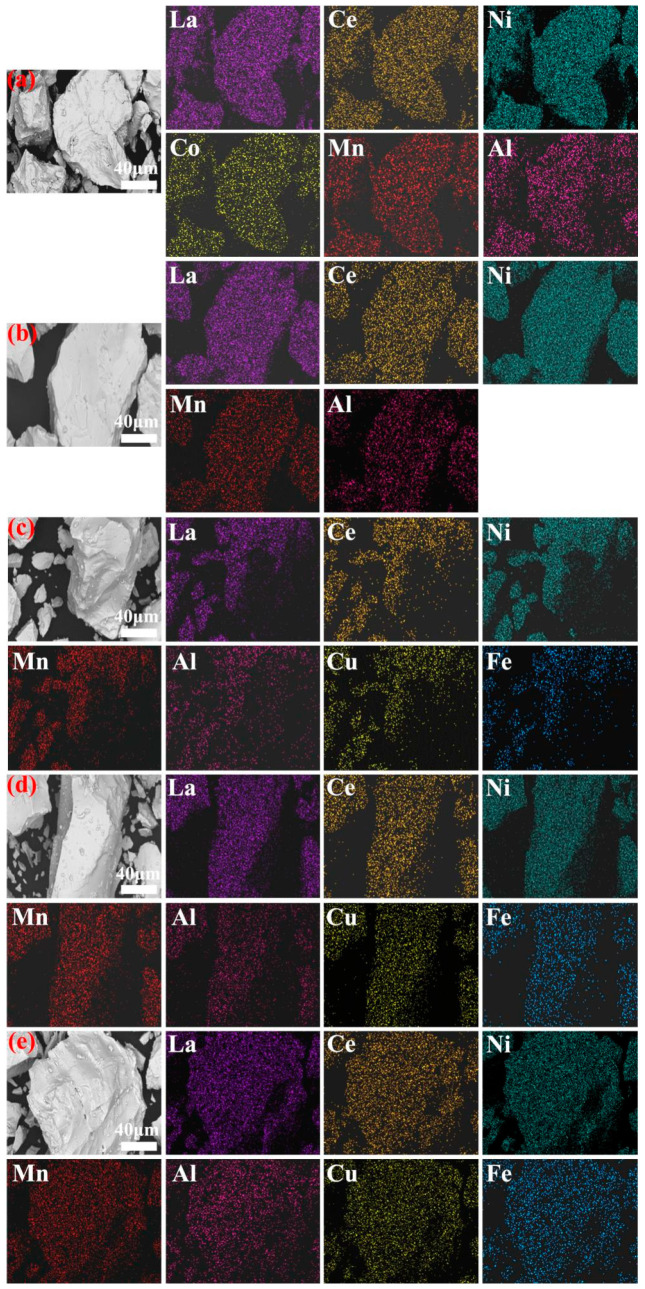
SEM images and EDS spectra of the hydrogen storage alloys: (**a**) alloy 4SC, (**b**) alloy 7D, (**c**) alloy 7L, (**d**) alloy 8A, and (**e**) alloy 8B.

To investigate the phase distribution and surface elemental composition of the alloys, EDS surface scanning tests and scanning electron microscopy were performed on the hydrogen storage alloys. Figure 2a–e display the SEM images and EDS spectra of all the alloys. It can be seen that the elements are uniformly distributed across the alloy surfaces. The alloy surfaces exhibit consistent coloration, indicating that the alloys are mono-phasic. These findings align with the XRD data.

### 3.2. PCT Test

**Figure 3:**
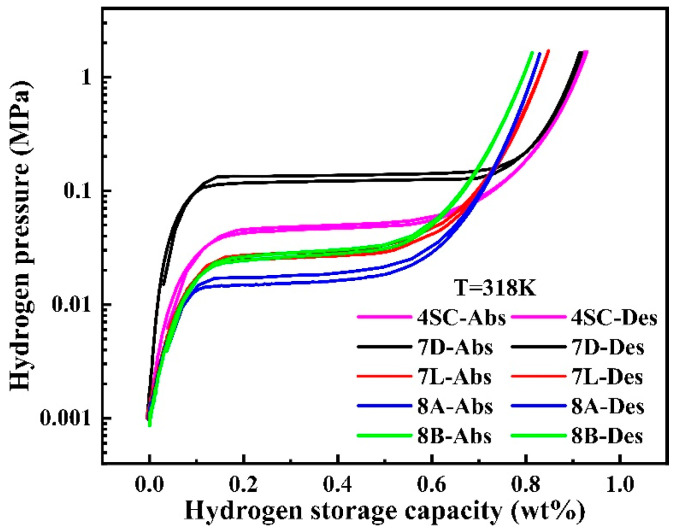
P-C-T curves for hydrogen absorption and desorption of hydrogen storage alloys.

Figure 3 depicts the PCT (Pressure–Composition–Temperature) isotherms of hydrogen sorption for hydrogen storage alloys. The detailed information is provided in Table 2. All alloys show moderate plateau pressures (0.001–0.1 MPa) and flat and extended plateaus. Alloys 4SC and 7D demonstrate longer plateaus, suggesting larger theoretical hydrogen storage capacities. In contrast, alloys 7L, 8A, and 8B, which are doped with varying amounts of Cu and Fe, exhibit lower plateau pressures, indicating higher stability of the hydride phase.

From Table 2, it is evident that the hydrogen storage capabilities of alloys 7L, 8A, and 8B exhibit lower values than those of alloys 4SC and 7D. When Cu and Fe elements substitute for Ni in the 3g positions of the LaNi_5_ alloy, their larger atomic radii occupy lattice sites that would otherwise accommodate hydrogen atoms, resulting in a reduction in the hydrogen storage capacity of the alloys. Among the alloys, alloy 7D exhibits the highest hydrogen sorption platform pressure. Using the unit cell volume parameters provided in Table 1, the results show that absorption platform pressure decreases as the unit cell size increases. This desorption platform stress follows a trend consistent with the absorption platform.

The slope (*F*) and hysteresis coefficient (*H_f_*) of the plateau are significantly higher for alloys 7L, 8A, and 8B doped with Cu and Fe. This phenomenon is mainly attributed to the replacement of Ni, which has a smaller atomic radius, with Cu and Fe with larger atomic radii. This substitution induces micro-strain and micro-stress within the alloy lattice, increasing the disorder of atomic arrangement. Consequently, the migration of hydrogen atoms into the lattice is hindered, leading to increased plateau slope and hysteresis.

### 3.3. Electrochemical Performance

#### 3.3.1. Maximum Discharge Capacity and Conventional Discharge Capacity

**Figure 4:**
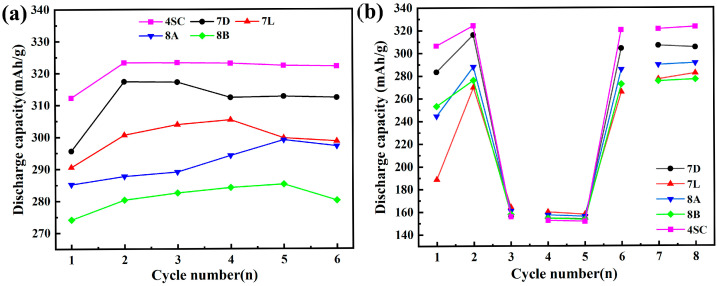
Electrochemical performance of hydrogen storage alloys: (**a**) Maximum discharge capacity. (**b**) Conventional capacity.

Figure 4 shows the maximum discharge capacity (a) and the conventional capacity (b) of the hydrogen storage alloys. The detailed information is provided in Table 3. As shown in Figure 4a and Table 3, alloys 4SC and 7D display superior activation behavior, reaching their peak discharge capacities between the second and third cycles under a current density of 0.2 C. In contrast, the other three alloys display poorer activation behavior. The peak discharge capacities of alloys 4SC, 7D, 7L, 8A, and 8B are 323.3 mAh g^−1^, 317.4 mAh g^−1^, 305.4 mAh g^−1^, 299.1 mAh g^−1^, and 311.2 mAh g^−1^, respectively. The presence of Co enhances activation performance, while Ni enhances discharge efficiency by destabilizing stable hydrides, thereby increasing the alloy’s capacity. Ni also exhibits high catalytic activity and excellent conductivity. Additionally, an increase in La content contributes to higher electrochemical capacity. Consequently, alloys 4SC and 7D exhibit superior activation and discharge capacity compared with the other alloys.

The conventional capacity method was employed to evaluate the average delivered capacity of the alloys. The eighth-cycle capacities for alloys 4SC, 7D, 7L, 8A, and 8B were determined using the rapid activation method, measuring 323.1 mAh g^−1^, 305.1 mAh g^−1^, 282.2 mAh g^−1^, 291.2 mAh g^−1^, and 276.8 mAh g^−1^, respectively, as depicted in Figure 4b. Traditional capacity data further demonstrate that the delivered capacity of alloys 4SC and 7D surpasses that of the other three alloys.

#### 3.3.2. Cycle Stability

**Figure 5:**
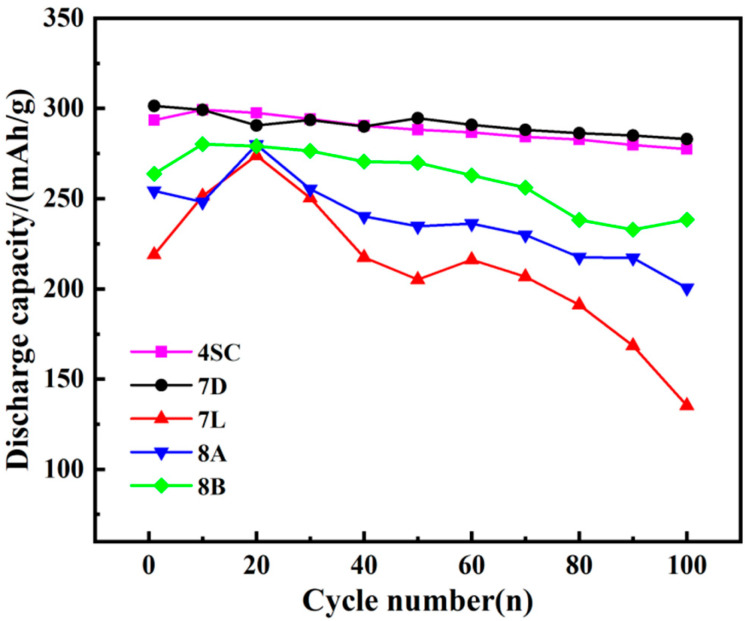
Cycle stability of hydrogen storage alloys.

The cycle stabilities of the hydrogen storage alloys are given in Figure 5. Combined with Table 3, it can be seen that alloy 7D demonstrates a capacity retention rate of 89.16% after 100 cycles, followed by alloy 4SC, with a retention rate of 85.83%. In contrast, the delivered capacity of alloy 7L shows a significant decline by the 100th charge–discharge cycle. Compared with the other four alloys, alloy 7D has higher Ni content. Ni exhibits excellent corrosion resistance in alkaline solutions, which contributes to extending the alloy’s service life. The delivered capacity of alloy 4SC following 100 cycles matches that of alloy 7D and outperforms the remaining three alloys. This is because the addition of Co reduces the micro-hardness of the hydrogen-absorbing alloy, thereby alleviating volume swelling following hydride formation and enhancing the alloy’s flexibility, ultimately improving its resistance to pulverization.

It is commonly recognized that oxidation and pulverization within alloy electrodes during charge–discharge cycles result in a decrease in discharge capacity [29]. The primary cause of pulverization in hydrogen storage alloys arises from unit cell volume swelling following hydrogen absorption, which fails to fully recover upon hydrogen desorption, leading to substantial internal stresses. Within the phase of the alloy, hydrogen exists as atoms occupying octahedral or tetrahedral interstitial sites in the alloy lattice. Throughout the charging procedure, hydrogen atom diffusion into the lattice induces lattice expansion, resulting in typical anisotropic characteristics. This leads to lattice deformation and generates significant internal stress. The lattice structure is disrupted by internal stress exceeding the critical threshold, leading to alloy pulverization. This pulverization increases the alloy’s specific surface area, thereby accelerating oxidation and corrosion. Both pulverization and oxidation are key factors contributing to the capacity degradation of the alloy.

Alloys 8A and 8B share the same elemental composition but differ in content, with the Fe content in alloy 8B exceeding that in alloy 8A. Since the atomic radius of Fe is greater than that of Cu, the unit cell volume increases, whereas the rate of lattice expansion decreases. This contributes to improved resistance to pulverization and an extended cycle life. Although alloy 7L contains the same elements as alloys 8A and 8B, it may possess excessive Mn content. The addition of Mn can enhance the specific electrochemical capacity of the alloy electrode. However, excessive Mn may lead to increased dissolution, causing surface oxidation and degrading the general performance of the alloy [30].

#### 3.3.3. High-Rate Properties

**Figure 6:**
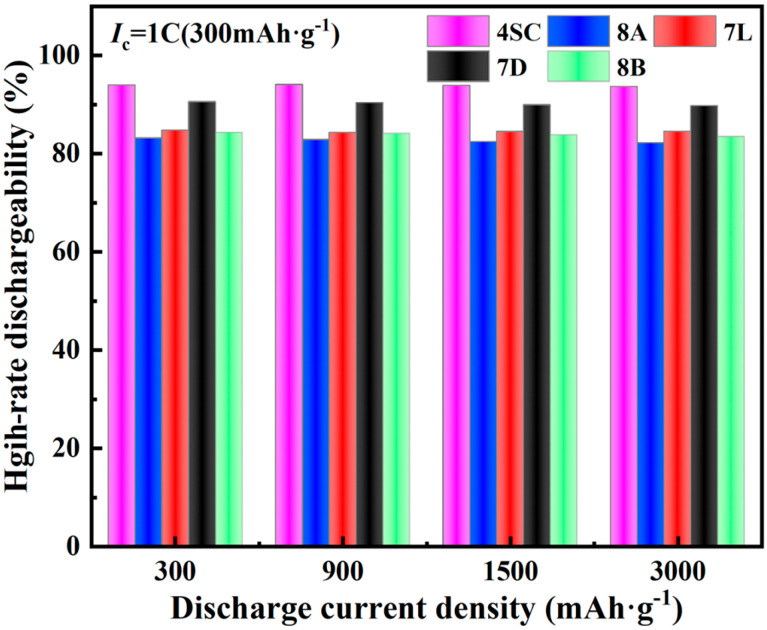
*HRD* performance of hydrogen storage alloys.

Figure 6 depicts the *HRD* performance of the hydrogen storage alloys. It is observed that the performance of the alloys shows minimal variation at different discharge rates. Notably, alloys 4SC and 7D exhibit more excellent *HRD* efficiencies of 94.1% and 90.3% at a 3 C rate, compared with the other three alloys; the discharge capacities were measured as 342.7 mAh g^−1^ and 328.8 mAh g^−1^, respectively. The *HRD* of alloys 7L, 8A, and 8B is comparatively inferior. Their discharge capacities were measured as 308.1 mAh g^−1^, 303.5 mAh g^−1^, and 305.4 mAh g^−1^, respectively. This difference may result from differences in Cu composition among these alloys. Cu forms a protective layer on the alloy phase surface, which adversely affects *HRD* and activation performance. These alloys also contain trace amounts of Fe. The partial substitution of Ni with a small amount of Fe reduces the c/a axial ratio, hindering the diffusion of H atoms into and egress from the lattice, thereby decreasing the *HRD* of the alloy. Additionally, this substitution may reduce the crystal defects in the alloy, thereby decreasing the migration paths for hydrogen atoms in the lattice and further lowering the *HRD* of the alloy.

### 3.4. Electrochemical Reaction Kinetics

Throughout the charging and discharging procedures of metal hydride electrodes, three key reactions occur:

M + H_2_O + e^−^ ⇔ MH_ad_ + OH^−^ (7)

MH_ad_ ⇔ MH_ab_ (8)

MH_ab_ ⇔ MH_hyd_ (9)

In the equations, *H*_ad_, *H*_ab_, and *H*_hyd_ denote H atoms adsorbed on the alloy surface, H atoms incorporated into the alloy lattice, and H atoms trapped as hydrides, respectively [31]. As depicted in Equations (7)–(9), throughout the charging process, the charge transfer process first takes place at the alloy particle–electrolyte interface. Subsequently, hydrogen atoms migrate into the alloy’s interior, ultimately forming metal hydrides. Typically, the electrode’s electrochemical kinetics are primarily dominated by these two steps [32]. The electrochemical kinetics of the alloy electrode in electrochemical reactions are affected not only by the charge transfer process at the alloy–electrolyte interface but also by the hydrogen diffusion rate within the alloy particles. The charge transfer process can be characterized via the charge transfer resistance (*R*_ct_) or the exchange current density (*I*_0_), whereas the hydrogen diffusion rate is quantified through the hydrogen diffusion coefficient (*D*). Thus, measuring these electrochemical kinetic parameters is critical to a comprehensive evaluation.

**Figure 7:**
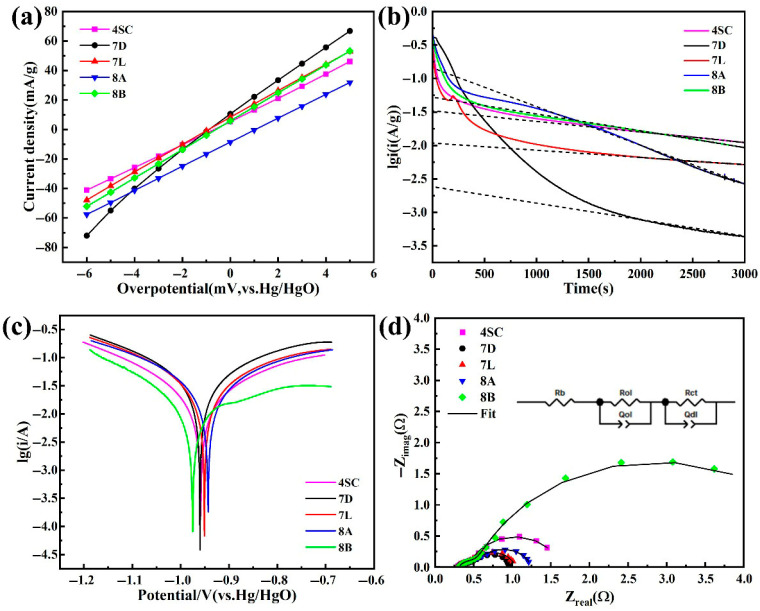
Electrochemical kinetics curves of hydrogen storage alloys: (**a**) Linear polarization curves. (**b**) Chronopotentiometric curves. (**c**) Tafel polarization curves. (**d**) EIS Nyquist plots.

Linear polarization and chronopotentiometric curves for the hydrogen storage alloys are presented in Figure 7a and Figure 7b, respectively. The fitted results are summarized in Table 4. After the addition of Cu and Fe to alloys 7L, 8A, and 8B, the exchange current density (*I*_0_) of the alloy electrodes is lower compared with that of alloys 4SC and 7D. This can be attributed to the elevated Ce amount within alloy 7D. The addition of Ce diminishes the stability of the metal hydride, thereby enhancing the diffusion rate of hydrogen atoms within the hydride phase. Thus, the general electrochemical kinetics of the alloy are enhanced. Interestingly, the hydrogen diffusivity (D) values of alloys 7D, 8A, and 8B are comparable, primarily because Cu, Fe, and Ni are neighboring elements with similar chemical properties within the same period of the periodic table.

Based on the equivalent circuit model proposed by Kuriyama et al. [33], the small semicircular arc in the high-frequency region represents the contact impedance between the electrode and the current collector, as well as among alloy particles, whereas the large semicircular arc in the mid-frequency region corresponds to the charge transfer resistance on the electrode surface. From the EIS Nyquist plots of electrochemical impedance spectroscopy (Figure 7d), it is apparent that the small semicircles of five hydrogen storage alloys are similarly sized, indicating that the contact resistance between alloy particles is nearly identical. In the mid-frequency range, semicircles with varying radii emerge. The larger semicircle corresponds to the alloy’s higher impedance, which decelerates the rate of charge transfer, thereby negatively impacting the alloy’s high-rate performance. *R*_ct_ represents the polarization resistance of electron migration at the alloy particle surface. In alloys 7D, 7L, and 8A, the exchange current density (*I*_0_) exhibits an inverse relationship with *R*_ct_, which is consistent with the *HRD* performance of the alloy electrodes. For alloy 4SC, the hybridization effect of Co in the hydrogen storage alloy accelerates hydrogen diffusion within the alloy, thus improving the charge–discharge efficiency. However, the addition of Co will also increase the plateau pressure of H absorption–desorption, which compromises the high-rate discharge performance. This also explains the reduction in its diffusion coefficient (*D*). Alloy 8B, with higher Cu content, demonstrates a comparable trend.

To further investigate the factors influencing the alloy’s cycle life, corrosion polarization curves were calculated, as depicted in Figure 7c. Table 4 presents the fitting results. In the Tafel polarization curve, a more positive corrosion potential indicates stronger corrosion resistance, while a smaller corrosion current corresponds to a slower corrosion rate of the alloy in alkaline solution. By analyzing the corrosion potential and current values (Table 4), it can be seen that among the three alloys doped with the same elements, alloys 7L and 8A exhibit superior corrosion resistance. This is primarily attributed to Cu addition, which enhances the alloy’s resistance to pulverization and improves its cycling stability. At the same time, the addition of Fe can also overcome the issues of alloy expansion and pulverization caused by hydrogen absorption. However, when combined with the cycling stability data, it is evident that alloys 4SC and 7D demonstrate better stability. This is because the effect of Cu in improving cycling performance is less pronounced than that of Co in alloy 4SC, while alloy 7D benefits from higher Ni content. Alloy 8B exhibits poor corrosion resistance due to the high Cu content, which adversely affects its cycling stability. This study focuses on five types of AB_5_ hydrogen storage alloys with varying element compositions and contents, aiming to explore the industrial application of such alloys. The five alloys investigated have all demonstrated commercial value, meeting different industrial requirements.

## 4. Conclusions

(1) XRD and SEM analyses reveal that after doping with various elements, the metal hydride alloys retain the single-phase CaCu_5_-type LaNi_5_ structure, with elements uniformly distributed throughout the alloy matrix.
(2) The electrochemical behavior of the metal hydride alloys was evaluated, revealing that alloys 4SC and 7D exhibit superior performance compared with the other three alloys. These two alloys attain their peak discharge capacities during the second or third cycle, with peak discharge capacities of 323.3 mAh g^−1^ and 317.4 mAh g^−1^, respectively. Their conventional discharge capacities are 323.1 mAh g^−1^ and 305.1 mAh g^−1^, respectively, and after 100 cycles, their capacity retention rates stay at 89.16% and 85.83%, respectively. Under a 3 C discharge rate, their *HRD* values are 94.1% and 90.3%, respectively.
(3) Kinetic performance analysis of the hydrogen storage alloys indicates that alloys 4SC and 7D exhibit outstanding performance, with I_0_ values of 302.97 mA g^−1^ and 317.70 mA g^−1^ and D values of 5.88 × 10^−10^ cm^2^ s^−1^ and 9.4 × 10^−10^ cm^2^ s^−1^, respectively. Cobalt-free alloy 7D demonstrates superior electrochemical kinetic performance compared with the other four alloys, providing a critical theoretical foundation for the development of cobalt-free industrial AB_5_-type hydrogen storage alloys.

## Figures and Tables

**Table 1:** Crystallographic parameters of the alloys.

Samples	Lattice Constants			Cell Volume (Å^3^)
	*a* (Å)	*c* (Å)	*c*/*a*	
4SC	5.0171	4.0512	0.8077	88.314
7D	5.0048	4.0459	0.8084	87.764
7L	5.0344	4.0871	0.8118	89.712
8A	5.0601	4.0780	0.8059	90.426
8B	5.0491	4.0797	0.8080	90.069

**Table 2:** Hydrogen storage capacity and plateau characteristics of hydrogen storage alloys at 318 K.

Samples	H_2_ Storage Capacity (wt%)	H_2_ Absorption Plateau (MPa)	H_2_ Desorption Plateau (MPa)	Slope	Hysteresis
4SC	0.926	0.050	0.047	3.928	0.062
7D	0.915	0.139	0.123	2.402	0.122
7L	0.848	0.029	0.026	4.638	0.109
8A	0.830	0.018	0.016	5.581	0.118
8B	0.814	0.030	0.027	5.314	0.105

**Table 3:** Electrochemical performance parameters of hydrogen storage alloys.

Samples	*N_a_* ^a^	*C_max_* (mAh g^−1^)	*C_r,_*_8_ ^b^ (mAh g^−1^)	*S*_100_ ^c^ (%)
		298 K		
4SC	3	323.3	323.1	85.83
7D	2	317.4	305.1	89.16
7L	4	305.4	282.2	44.30
8A	5	299.1	291.2	67.03
8B	5	311.2	276.8	76.57

^a^ The number of cycles required to achieve the alloys’ maximum discharge capacity at a 0.2 C rate and 298 K. ^b^ The average discharge capacity of the alloy at the eighth cycle, determined using the conventional capacity testing method. ^c^ The capacity retention measure value *S*_100_ is characterized as the proportion of the delivered capacity after 100 charge–discharge cycles to the maximum capacity at a 1 C rate and 25 °C.

**Table 4:** Kinetic parameters of hydrogen storage alloys.

Samples	R_ct_ (Ω)	*I*_0_ (mA/g)	*D* (×10^−10^ cm^2^/s)	φ_corr_ (V)	*i*_corr_ (A)
4SC	0.875	302.97	5.88	−0.961	0.011
7D	0.490	317.70	9.45	−0.961	0.021
7L	0.580	235.39	4.04	−0.952	0.017
8A	0.646	209.46	9.36	−0.944	0.016
8B	4.703	246.50	9.36	−0.987	0.010

## Data Availability

The original contributions presented in this study are included in the article. Further inquiries can be directed to the corresponding author.
